# A combination of serum leucine-rich *α*-2-glycoprotein 1, CA19-9 and interleukin-6 differentiate biliary tract cancer from benign biliary strictures

**DOI:** 10.1038/bjc.2011.376

**Published:** 2011-10-04

**Authors:** N S Sandanayake, J Sinclair, F Andreola, M H Chapman, A Xue, G J Webster, A Clarkson, A Gill, I D Norton, R C Smith, J F Timms, S P Pereira

**Affiliations:** 1 UCL Institute of Hepatology, University College London, 9th Floor Royal Free Hospital, Pond Street, London NW3 2PG, UK; 2 Cancer Proteomics Laboratory, EGA Institute for Women's Health, University College London, Gower Street, London, WC1E 6BT, UK; 3 Kolling Institute, University of Sydney, Royal North Shore Hospital, Reserve Road, St Leonards, New South Wales 2065, Australia; 4 Department of Gastroenterology, University College Hospitals NHS Foundation Trust, 235 Euston Road, London, NW1 2BU, UK; 5 Department of Anatomical Pathology, Royal North Shore Hospital, Reserve Road, St Leonards, New South Wales 2065, Australia; 6 Department of Gastroenterology, Royal North Shore Hospital, Reserve Road, St Leonards, New South Wales 2065, Australia

**Keywords:** biliary tract cancer, PSC, IAC, serum proteomic profiling, leucine-rich *α*-2-glycoprotein, interleukin-6

## Abstract

**Background:**

Biliary tract cancer (BTC) and benign biliary strictures can be difficult to differentiate using standard tumour markers such as serum carbohydrate antigen 19-9 (CA19-9) as they lack diagnostic accuracy.

**Methods:**

Two-dimensional difference gel electrophoresis and tandem mass spectrometry were used to profile immunodepleted serum samples collected from cases of BTC, primary sclerosing cholangitis (PSC), immunoglobulin G4-associated cholangitis and healthy volunteers. The serum levels of one candidate protein, leucine-rich *α*-2-glycoprotein (LRG1), were verified in individual samples using enzyme-linked immunosorbent assay and compared with serum levels of CA19-9, bilirubin, interleukin-6 (IL-6) and other inflammatory markers.

**Results:**

We report increased LRG1, CA19-9 and IL-6 levels in serum from patients with BTC compared with benign disease and healthy controls. Immunohistochemical analysis also demonstrated increased staining of LRG1 in BTC compared with cholangiocytes in benign biliary disease. The combination of receiver operating characteristic (ROC) curves for LRG1, CA19-9 and IL-6 demonstrated an area under the ROC curve of 0.98. In addition, raised LRG1 and CA19-9 were found to be independent predictors of BTC in the presence of elevated bilirubin, C-reactive protein and alkaline phosphatase.

**Conclusion:**

These results suggest LRG1, CA19-9 and IL-6 as useful markers for the diagnosis of BTC, particularly in high-risk patients with PSC.

Cholangiocarcinoma (CCA) and gallbladder carcinoma are often grouped together as biliary tract cancer (BTC) (de Groen *et al*, 1999). CCA is a rare but devastating disease that accounts for ∼15% of primary liver malignancies (Shaib and El-Serag, 2004). Although the incidence in Western nations has been estimated at 1 per 100 000, there are as yet unexplained increases in the age-adjusted incidence and mortality rates of intrahepatic CCA (Khan *et al*, 2002b; Shaib *et al*, 2004).

Disorders of the bile ducts associated with inflammation, stricturing and cholestasis, such as primary sclerosing cholangitis (PSC), have been identified as risk factors for BTC (Broome *et al*, 1995; Kornfeld *et al*, 1997; Bergquist *et al*, 2002; Burak *et al*, 2004; Claessen *et al*, 2009). Other inflammatory biliary diseases such as immunoglobulin G4-associated cholangitis (IAC) are also difficult to distinguish from BTC and PSC, occasionally leading to incorrect diagnosis and management. At present, surgical resection is the only curative treatment for BTC, with 5-year survival rates after R0 resection for hilar CCA of 11–41% and for distal extrahepatic CCA of 27–37% (Nakeeb *et al*, 1996; Neuhaus *et al*, 1999; Jarnagin *et al*, 2001; Nagorney and Kendrick, 2006; DeOliveira *et al*, 2007). Unresectable disease has an associated 5-year survival of less than 10% (Neuhaus *et al*, 1999; Reddy and Patel, 2006), and confirms the need for more accurate diagnostic biomarkers.

The gold standard for the diagnosis of BTC is cytological or histological confirmation of malignancy within the biliary stricture, either via endoscopic retrograde cholangiopancreatography (ERCP) or percutaneously (Khan *et al*, 2002a). However, this can be difficult to achieve due to the complex anatomical relationship between the liver hilum and bile ducts and the mode of tumour extension, which is often not mass forming. Approximately 15% of resections performed for suspected, pathologically unconfirmed, hilar BTC are due to benign strictures (Gerhards *et al*, 2001; Reddy and Patel, 2006).

Carbohydrate antigen 19-9 (CA19-9) is an established serum marker for the diagnosis of BTC, although it is reported to have a wide variation in sensitivity (50–90%) and specificity (54–98%), (Chalasani *et al*, 2000; Patel *et al*, 2000; Levy *et al*, 2005) and is often falsely elevated in benign biliary disease and/or cholangitis, with levels falling after relief of biliary obstruction and sepsis. Additionally, CA19-9 is virtually undetectable in the 7% of the population who are negative for the Lewis antigen (Steinberg, 1990). There is consequently a need to identify better circulating biomarkers for this disease. However, tumour marker proteins may be present at low concentrations in the circulation and masked by abundant blood proteins in proteomic analyses. Consequently, researchers have coupled immunoaffinity depletion with proteomic profiling to specifically remove abundant proteins and increase proteomic coverage (Liu *et al*, 2006; Qian *et al*, 2008).

This study analysed differential protein expression in four different pools of serum from patients with BTC, PSC, IAC and healthy volunteers. Using proteomic analysis the putative marker leucine-rich *α*-2-glycoprotein (LRG1) was shown to be upregulated in BTC, and was validated using a serum enzyme-linked immunosorbent assay (ELISA). The ability of LRG1 to differentiate BTC from benign biliary disease was evaluated and compared with the current standard tumour marker CA19-9, while taking into consideration potentially confounding conditions such as biliary obstruction and inflammation. Immunohistochemistry for LRG1 was performed on normal hepatobiliary tissue, benign biliary disease (PSC and primary biliary cirrhosis (PBC)) and BTC to evaluate differential staining characteristics. Serum interleukin-6 (IL-6) was also measured as it has been recently shown to be a key inflammatory cytokine involved in the carcinogenesis of CCA and may induce LRG1 (Hodge *et al*, 2005; Shirai *et al*, 2009).

## Patients and methods

### Patient population and clinical samples

The study was conducted following local ethical approval (06/Q0152/106) and written informed consent. Blood samples were prospectively collected from patients with BTC (*n*=37), PSC (*n*=11), IAC (*n*=7) and healthy volunteers (*n*=30) using Vacutainer tubes (BD, Franklin Lakes, NJ, USA). Primary sclerosing cholangitis was diagnosed on the basis of well-described clinical characteristics, primarily based on cholangiographic presence of biliary strictures and benign cytological biliary brushings, and where possible, supportive evidence such as the presence of inflammatory bowel disease or liver histology consistent with the diagnosis (Chapman *et al*, 1980). A diagnosis of IAC was based on the HISORt (HIstology, Serology and Imaging, Other organ involvement and Response to therapy) criteria (Chari, 2007). The clinical and pathological characteristics of the BTC patients who had stricturing ductal cancers rather than mass-forming tumours are presented in Table 1 and in Supplementary Information.

Baseline patient demographics and standard blood tests including liver biochemistry (bilirubin and alkaline phosphatase (ALP)), white cell count (WCC), neutrophil count, C-reactive protein (CRP), IgG4 and CA19-9 (electro-chemiluminescence immunoassay; Roche Modular, Indianapolis, IN, USA) were recorded at the time blood was taken (see Table 2). Blood samples for biochemical analysis were sent to the hospital laboratory for immediate processing, whereas for proteomic analysis, samples were allowed to clot at room temperature for 1 h, and then separated by centrifugation at 2200 r.p.m. for 10 min at 4 °C. Serum was decanted, aliquoted and stored at −80 °C. For proteomic analysis, all samples were thawed and an equal volume of each was pooled into one of four groups; BTC, PSC, IAC and healthy volunteers. The individual pools were vortexed to ensure thorough mixing, then aliquoted into cryovials and stored at −80 °C.

### FPLC immunoaffinity depletion using tandem IgY14-Supermix system

IgY14 (12.7 × 39.5 mm, Affinity Column, Seppro (GenWay Biotech, San Diego, CA, USA), 28-288-12014-LC5) and SuperMix (6.4 × 31.5 mm, Affinity Column, Seppro GenWay Biotech, 28-288-23078-LC1) columns were used in series on a cooled AKTA FPLC system (GE Healthcare, Piscataway, NJ, USA) to deplete the serum pools of abundant proteins following the manufacturer's recommendations, and were similar to those described previously (Qian *et al*, 2008) (described in Supplementary Information).

### Sample desalting, concentration and preparation

The protein-depleted serum was concentrated using 4-ml 5 kDa molecular weight cutoff Vivaspin centrifugal concentrators (Sartorius, Goettingen, Germany) at 3200 g and 10 °C, the buffer was exchanged with distilled water and further concentrated before drying down in a SpeedVac (Thermo Scientific, Waltham, MA, USA). Samples were resuspended in 100 *μ*l of 2D lysis buffer (8 M urea, 2 M thiourea, 4% CHAPS, 0.5% IGEPAL, 10 mM Tris, pH 8.3) and protein concentrations determined using the Coomassie Plus protein assay (Pierce, Rockford, IL, USA). To evaluate the effectiveness of the immuno-depletion, samples were analysed by 1D-SDS-PAGE on NuPAGE Novex 4–12% Bis-Tris 1.5 mm × 10 well pre-cast gels (Invitrogen, Carlsbad, CA, USA) and stained with InstantBlue (Expedeon, Cambridge, UK).

### Two-dimensional difference gel electrophoresis

For protein labelling, the N-hyroxysuccinimidyl ester cyanine dyes NHS-Cy3 and NHS-Cy5 were synthesised ‘in-house’ as previously described (Chan *et al*, 2005). Protein labelling and two-dimensional difference gel electrophoresis (2D-DIGE) were performed according to (Gharbi *et al*, 2002) and is described more detail in Supplementary Information. Briefly, 100 *μ*g each of triplicate immuno-depleted protein pools were labelled with NHS-Cy3 or NHS-Cy5. Equal amounts of protein from all fractions were also pooled together and labelled with NHS-Cy2 (GE Healthcare, Piscataway, NJ, USA) as an internal standard run on all gels against the pairs of Cy3- and Cy5-labelled samples to aid in spot matching and for improved quantitative accuracy. Proteins were separated in the first dimension using isoelectric focusing, with 24 cm, non-linear pH 3-10 IPG strips (GE Healthcare), and in the second dimension on 1.5 mm 12% SDS-PAGE bonded gels. Following scanning and analysis using DeCyder software V5.0 (GE Healthcare), protein spots displaying a ⩾1.5 average-fold change in abundance between clinical conditions with *P*-values <0.05 were selected for picking on an Ettan automated spot picker (GE Healthcare).

### Protein identification by LC-MS/MS

Protein spots were subjected to trypsin digestion and peptide extraction according to a previous protocol (Chan *et al*, 2005). Liquid chromatography-tandem mass spectrometry (LC-MS/MS) was performed using an Ultimate 3000 nanoflow liquid chromatography system (Dionex, Sunnyvale, CA, USA) coupled via a Picoview nanospray source (New Objective, Inc., Woburn, MA, USA) to an LTQ Orbitrap XL mass spectrometer (Thermo Scientific, Waltham, MA, USA) as previously described (Sinclair *et al*, 2011). For peptide identification, raw data files produced in Xcaliber software (Thermo Scientific) were transferred to Mascot Daemon for processing by Mascot Distiller V2.2.02, where peak detection was carried out for searching against the IPI human database (v3.53, 73 748 entries) (described in detail in Supplementary Information). Literature searches were carried out to determine previous associations between the identified proteins and cancer, and more specifically BTC. On the basis of this, LRG1 was chosen for further confirmation of differential expression.

### LRG1 and IL-6 ELISA

The levels of LRG1 in undepleted serum samples from a subset of individual BTC patients (*n*=31), PSC/IAC patients (*n*=11/2) and healthy volunteers (*n*=15) were evaluated using a commercially available quantitative human LRG1 ELISA Assay Kit (Immuno-Biological Laboratories Co Ltd, Takasaki, Gunma, Japan). The assay gave a CV of 5.5% when tested on replicate samples used in this study. Serum IL-6 levels were evaluated using a commercially available quantitative human IL-6 ELISA Assay Kit (Pierce) in a subset of BTC patients (*n*=24), PSC/IAC patients (*n*=10/2) and healthy volunteers (*n*=6) and gave a CV of 6.7%.

### LRG1 immunohistochemistry

Immunohistochemistry for LRG1 was performed on formalin-fixed paraffin-embedded tissues from one normal liver, one gallbladder with mild chronic cholecystitis, two liver biopsies showing PSC and PBC, and two CCA specimens using a commercially available affinity-purified rabbit polyclonal antibody, at a dilution of 1 : 1000 (13224-1-AP, ProteinTech, Chicago, IL, USA) (described in detail in Supplementary Information).

### Statistical analysis

Statistical analyses were carried out using the GraphPad Prism V5 (La Jolla, CA, USA) and SPSS v10 (Chicago, IL, USA) software packages. All sample size calculations were performed using an *α* of 0.05 and *β* of 0.80 in MedCalc v11.4.2 (Mariakerke, Belgium). Continuous data between clinical groups were compared using the Mann–Whitney *U*-test for non-parametric data, and the Student's *t*-test for normally distributed data. Spearman's rank correlation was used to examine associations between two continuous variables. Associations between categorical variables were examined using Fisher's exact test. Multiple logistic regression analysis was used to examine the inter-relationships between serum proteins, biliary obstruction, patient age and cancer likelihood. As the CA19-9, bilirubin, CRP and ALP data were skewed, logarithmic transformation was applied before carrying out regression analyses. Normal cutoffs were defined for LRG1 (57.5 *μ*g ml^−1^) and IL-6 (48.4 pg ml^−1^) as the optimum point at which sensitivity and specificity was maximised, whereas a cutoff of 37 U ml^−1^ for CA19-9 was recommended by the manufacturer. The sensitivity, specificity, positive and negative predictive values and accuracy were calculated for all three markers. Receiver operating characteristic (ROC) curves were generated and the area under the ROC curves (area under the curve (AUC)) defined. The combined AUC for a panel of biomarkers was calculated to define the diagnostic accuracy of discriminating benign from malignant biliary strictures. A *P*-value of <0.05 was considered significant.

## Results

### Identification of differentially expressed serum proteins in immuno-depleted fractions using 2D-DIGE/MS

**Figure 1:**
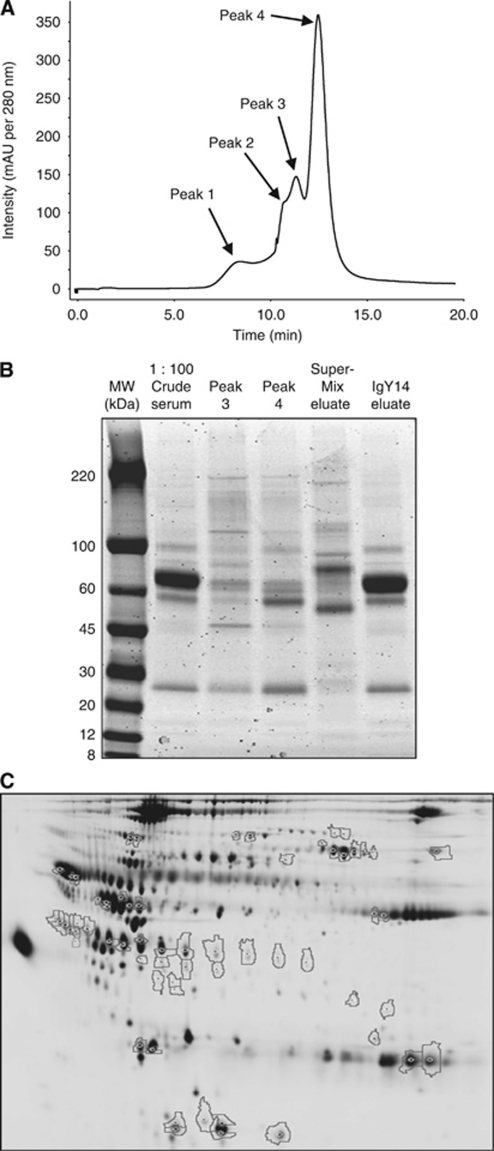
(**A**) Liquid chromatogram of flow-through fractions following immunoaffinity binding of high- and medium-abundance serum proteins. Protein peaks 1 to 4 were separately collected for analysis. (**B**) 1D-SDS PAGE and protein staining of flow-through peaks 3 and 4 obtained by FPLC immunoaffinity serum depletion confirming efficient depletion of high-abundance proteins. Diluted crude serum (1 : 100) and eluates from both IgY14 and SuperMix columns were run on the gel for comparison. A volume of 4.3 *μ*g protein was loaded per lane. (**C**) Master gel image from 2D-DIGE experiment. The Cy2-labelled internal standard is shown. Highlighted spots were consistently differentially expressed between two or more of the clinical groups.

Immunodepletion using FPLC over three or four runs for each of the four clinical pools were consistent, as were protein yields over all runs (Supplementary Information, Supplementary Figures S1 and S2). The depleted flow-through fractions contained four peaks, which were collected separately and protein contents compared with crude serum and eluates from the two columns (obtained by acid stripping) by 1D-SDS-PAGE with colloidal Coomassie Blue staining (Figures 1A and B). Protein bands were observed in the flow-through peak lanes that were not detected in both crude serum and column eluates, demonstrating the depletion of abundant proteins.

Immuno-depleted fractions were analysed by 2D-DIGE in triplicate to identify proteins whose expression differed significantly between sample groups. Image analysis revealed ∼1500 protein spots with protein abundance differences of >1.5-fold (*P*<0.05) considered as proteins of interest (Figure 1C). A total of 56 protein spots were altered between BTC and healthy volunteers, 47 between BTC and PSC and 31 spots between PSC and IAC. Spots of interest were excised from SyproRuby (Molecular Probes, Inc., Eugene, OR, USA) post-stained DIGE gels and subjected to LC-MS/MS-based analysis and database searching for identification. A total of 133 high-confidence protein identifications were made with many of the spots containing multiple proteins, as expected (Supplementary Information, Supplementary Table S1). Several proteins with a potential role in carcinogenesis were identified, including LRG1 and tetranectin/CLEC3B (Table 3). Isoforms of LRG1 were found in seven separate gel spots, all significantly upregulated in BTC *vs* healthy, PSC and IAC pools. One form found as a single identification in one gel spot was upregulated 4.49-fold in the BTC *vs* healthy pools, 3.57-fold in BTC *vs* PSC and 2.95-fold in BTC *vs* IAC. The altered expression of LRG1 was thus further examined in individual serum samples using a commercial ELISA.

### Confirmation of differential expression of LRG1

**Figure 2:**
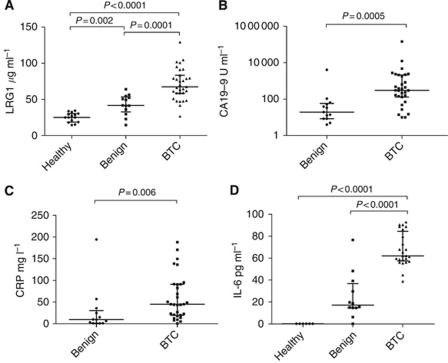
Scatter plots of serum levels of (**A**) LRG1, (**B**) CA19-9, (**C**) CRP in samples from BTC patients (*n*=31), PSC/IAC patients (*n*=11/2) and healthy volunteers (*n*=15) used in the LRG1 validation experiment. (**D**) Scatter plot of serum IL-6 levels in a subgroup of BTC patients (*n*=24), benign biliary disease (PSC *n*=10, IAC *n*=2) and healthy volunteers (*n*=6) showing significant differences between groups. *P*-values (Mann–Whitney *U*-test) are shown.

To validate the 2D-DIGE result for LRG1 expression, an ELISA was run using serum from a subset of 59 individual patients that were used for the original pools. Figure 2A demonstrates that LRG1 levels were significantly elevated in the BTC group (median 67.4 *μ*g ml^−1^, inter-quartile range (IQR) 58.6–83.4 *μ*g ml^−1^) when compared with either the benign (PSC/IAC) (median 41.8 *μ*g ml^−1^, IQR 32.9–53.4 *μ*g ml^−1^; *P*=0.0001) or healthy volunteer groups (median 25.0 *μ*g ml^−1^, IQR 18.7–30.4 *μ*g ml^−1^; *P*<0.0001). Unlike the values for CA19-9 and serum bilirubin, LRG1 values were centrally distributed with skewness <0.5 (Figures 2A and B). The serum levels of LRG1 in individual patients belonging to the three clinical groups were thus in concordance with the 2D-DIGE analysis of pooled groups of samples.

### Association between LRG1, CA19-9, biliary obstruction, raised inflammatory markers and likelihood of BTC

The BTC group had significant elevations of median CA19-9 (*P*=0.0005), bilirubin (*P*=0.04), CRP (*P*=0.006), ALP (*P*=0.0002) and neutrophil count (*P*=0.02) compared with the benign group, but not for WCC (Table 4, Figure 2 and data not shown). Serum bilirubin was elevated (>21 *μ*mol l^−1^) in 24/31 BTC patients and 4/13 PSC/IAC patients. Serum LRG1 was positively correlated with bilirubin (Spearman's *ρ*=0.303, *P*=0.045) and ALP levels (*ρ*=0.670, *P*<0.0001). A significant correlation was also observed between LRG1 and CRP (*ρ*=0.687, *P*<0.0001), neutrophil count (*ρ*=0.541, *P*<0.0001) and WCC (*ρ*=0.455, *P*=0.002). Logistic regression analysis was undertaken to assess the independence of the association between LRG1 and likelihood of BTC from the effects of obstructive jaundice and inflammation. Given the skewed distribution of CA19-9, bilirubin, ALP and CRP, levels were normalised for subsequent regression analyses by logarithmic transformation. When LRG1, log_10_CA19-9, log_10_CRP, log_10_ALP and log_10_bilirubin were included on a univariate logistic regression model, all factors except log_10_bilirubin were independently associated with BTC. On multivariate analysis, only increased levels of LRG1 (odds ratio (OR)=1.09 (95% CI: 1.02–1.17); *P*=0.017) and CA19-9 (OR=4.24 (95% CI: 1.19–15.10); *P*=0.026) continued to exhibit an independent association with BTC likelihood. Patient age was a confounding factor for LRG1, with increasing age being significantly correlated with higher levels of LRG1 (*ρ*=0.345, *P*=0.022). Given the reported involvement of the inflammatory cytokine IL-6 in the carcinogenesis of CCA and its ability to induce LRG1, serum IL-6 levels were also measured in a subset of the samples. Median IL-6 levels were 62.1 pg ml^−1^ (IQR, 57.5–84.6 pg ml^−1^) for the BTC group, 17.1 pg ml^−1^ (IQR, 14.6–36.8 pg ml^−1^) for the benign group and <0.2 pg ml^−1^ for the healthy controls. Thus, IL-6 levels were significantly higher in BTC *vs* both the benign and healthy volunteer groups (*P*<0.0001) (Figure 2F). LRG1 was also positively correlated with serum IL-6 level (Spearman, *ρ*=0.631, *P*<0.001).

### ROC curve analysis and diagnostic performance

**Figure 3:**
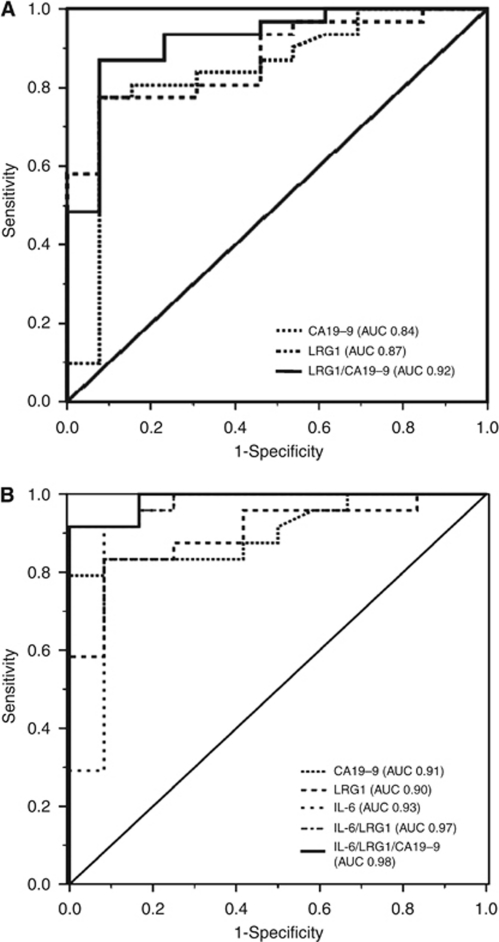
ROC curves and AUC for the following: (**A**) CA19-9, LRG1 and CA19-9/LRG1 combined; (**B**) CA19-9, LRG1, IL-6 and CA19-9/LRG1/IL-6 combined, in discriminating BTC from benign biliary disease.

The ROC curve AUC for LRG1 and CA19-9 were 0.87 (95% CI: 0.77–0.98) and 0.84 (95% CI: 0.69–0.98), respectively, whereas their combination was 0.92 (*P*<0.001) (Figure 3A). The AUC for serum IL-6 was 0.93 (95% CI 0.82–1.0). The AUC for the combination of LRG1 and IL-6 ROC curves was 0.97 (*P*=0.03). Finally, the combination of ROC curves for LRG1, CA19-9 and IL-6 gave an AUC of 0.98 (*P*=0.01) (Figure 3B). The sensitivity, specificity, positive predictive value, negative predictive value and accuracy for LRG1 (cutoff >57.5 *μ*g ml^−1^) in distinguishing malignant from benign biliary strictures were 83, 92, 95, 73 and 86%, respectively. The corresponding figures for CA19-9 (cutoff >37 U ml^−1^) and IL-6 (cutoff >48.4 pg ml^−1^) were 83, 67, 83, 67 and 78% and 92, 92, 96, 85 and 92%, respectively.

### LRG1 immunohistochemistry

**Figure 4:**
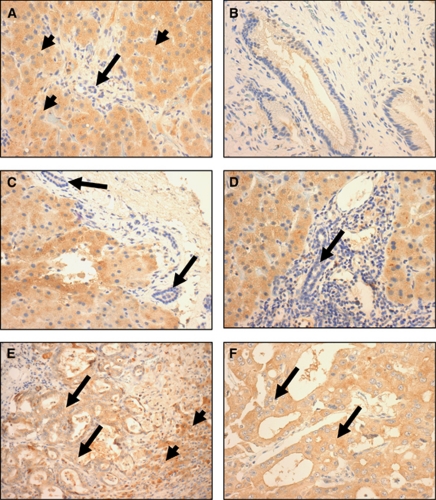
LRG1 immunohistochemistry analysis demonstrating: (**A**) moderate expression of LRG1 in normal liver (arrowheads) with no expression in biliary epithelium (arrow) (original magnification × 400), (**B**) no expression of LRG1 in normal biliary epithelium from gallbladder (original magnification × 400), (**C**) PSC showing positive staining of hepatocytes with no staining of the biliary epithelium (arrow) (original magnification × 400), (**D**) PBC showing positive staining of hepatocytes with no staining of the biliary epithelium (arrow) (original magnification × 400), (**E**). cholangiocarcinoma showing positive expression in malignant cells (arrow) and in (arrow) adjacent non-neoplastic hepatocytes of the liver (arrowheads) (original magnification × 200), (**F**) cholangiocarcinoma showing positive expression of LRG (original magnification × 400).

The pattern of LRG1 protein expression was assessed by immunohistochemistry in morphologically normal, benign and malignant hepatobiliary resection tissue. In normal liver, bile ducts did not stain for LRG1, whereas hepatocytes showed weak to moderate staining (Figure 4A). Biliary epithelium from a gallbladder showing chronic cholecystitis demonstrated negative staining for LRG1 (Figure 4B). In the PSC (Figure 4C) and PBC (Figure 4D) liver biopsies the biliary epithelium was negative for LRG1, but there was positive staining of hepatocytes, which was most marked in the periportal zones and greater than that seen in normal livers. Two CCAs (Figures 4E and F) showed positive staining for LRG1, both in the malignant cells and non-neoplastic hepatocytes in the liver adjacent to the carcinomas, most marked in the periportal zones.

## Discussion

Recent applications of proteomic technologies have identified putative serum markers in a variety of gastrointestinal malignancies (Lin *et al*, 2009). However, relatively few proteomic studies of BTC using cell lines, bile or tissue have been performed, and only two have investigated circulating markers (Scarlett *et al*, 2006; Wang *et al*, 2009). In this rare cancer (Shaib and El-Serag, 2004), our discovery analysis of pooled serum samples using immunoaffinity depletion, 2D-DIGE and LC-MS/MS was able to detect differential protein expression between the BTC, benign and healthy groups, including upregulation of the putative marker LRG1. This upregulation was further verified in individual samples using an ELISA, which confirmed significant elevation of serum LRG1 in BTC compared with benign biliary disease (PSC and IAC) and healthy volunteers.

In hepatobiliary cancer, an ideal tumour marker should distinguish malignant from benign disease in the context of confounding factors such as obstructive jaundice and inflammation. In clinical practice, bile duct stenoses in BTC can be difficult to differentiate from those secondary to PSC and IAC (Chapman, 1999), as patients may share similar clinical, biochemical and radiological characteristics. Diagnosing CCA in patients with underlying PSC-related strictures using ERCP-guided biliary brush cytology or endoscopic ultrasound-guided fine needle aspiration can be challenging as sensitivity and specificity for brush cytology is inconsistent, and mass lesions are uncommon in early CCA. The positive predictive value of ultrasound, computed tomography and magnetic resonance imaging in identifying cancer in these PSC patients is also limited (Charatcharoenwitthaya *et al*, 2008). Recent studies of PSC have reported a 7–9% 10-year cumulative incidence of developing BTC (Burak *et al*, 2004; Claessen *et al*, 2009), and a lifetime risk of 20% (Bergquist *et al*, 2002; Burak *et al*, 2004). However, this may be an underestimation of the true prevalence, because the occurrence of CCA in autopsies or explanted livers of PSC patients undergoing transplantation maybe as high as 30% (Broome *et al*, 1996; Chalasani *et al*, 2000). These factors underline the need for effective markers of BTC in these high-risk groups.

Biliary obstruction and cholestatic liver enzymes can confound the significance of protein biomarkers in the context of pancreaticobiliary cancer (Yan *et al*, 2009). Here, by multivariate logistic regression, LRG1 was shown to be an independent predictor of BTC in the presence of elevated bilirubin, ALP and CRP. Carbohydrate antigen 19-9 was also an independent predictor of cancer. An LRG1 level of 57.5 *μ*g ml^−1^ had a positive predictive value of 95% in discriminating BTC from benign biliary strictures, highlighting its potential use as a screening tool in PSC patients. The AUC for LRG1 was similar to that for CA19-9 (0.87 *vs* 0.84), whereas the combination of the two markers yielded a significantly higher (*P*<0.001) AUC of 0.92 compared with either marker alone. However, it should be emphasised that the sample size – particularly in the cohort with benign biliary strictures – was relatively small, so that these promising results will require validation in a larger sample set.

To minimise the likelihood of elevated acute-phase response proteins being due to infection, patients with symptoms or signs of ascending cholangitis were excluded from the study. Any elevation of CRP and IL-6 was thus interpreted to reflect inflammation secondary to benign or malignant biliary disease, rather than infection. The inflammatory cytokine IL-6 is increased in the biliary tract and systemic circulation in patients with CCA (Goydos *et al*, 1998; Sugawara *et al*, 1998; Tangkijvanich *et al*, 2004; Cheon *et al*, 2007), and has been proposed as a crucial cytokine involved in the pathogenesis of CCA (Okada *et al*, 1994; Park *et al*, 1999; Yokomuro *et al*, 2000; Wehbe *et al*, 2006; Yamagiwa *et al*, 2006; Isomoto *et al*, 2007). Recent work has also targeted IL-6 for the development of novel therapies for CCA (Braconi *et al*, 2011). Furthermore, the induction of LRG1 by IL-6 has been shown to be upregulated synergistically with mediators of inflammation such as TNF*α* in cell culture (Shirai *et al*, 2009). We thus assessed the utility of serum IL-6 levels in differentiating malignant from the benign inflammatory biliary diseases PSC and IAC. We showed a significant increase in serum IL-6 levels in BTC compared with benign biliary disease and healthy volunteers, and a positive correlation with LRG1 in BTC patients. A combination of the ROC curves for LRG1, IL-6 and CA19-9 yielded an AUC of 0.98 for the diagnosis of BTC *vs* PSC/IAC, supporting the concept that a combination of these markers may be an appropriate strategy for differential diagnosis in the clinic.

On immunohistochemical analysis, LRG1 expression was demonstrated in hepatocytes from normal livers with overexpression in hepatocytes, but not biliary epithelium, in the PSC and PBC benign cholestatic conditions. In CCA there was overexpression in malignant biliary epithelium in addition to increased hepatocyte staining, which may therefore account for the observed increase in LRG1 serum levels in BTC patients compared with PSC/IAC patients and healthy volunteers. Although the immunohistochemistry was performed on a small number of samples and is thus preliminary, the data suggest the potential of LRG1 staining to differentiate benign and malignant strictures in cytological brushings obtained at ERCP.

The physiological function of LRG1 is not known. LRG1 has been previously detected in the bile of a CCA patient using proteomic methods (Kristiansen *et al*, 2004), suggesting that it may be a glycoprotein which is synthesised in the liver and undergoes biliary excretion. Elevated plasma LRG1 has also been reported in patients with pancreatic cancers (Kakisaka *et al*, 2007), though its relationship to the presence or absence of biliary obstruction in these patients was not reported.

Given the rarity of BTC, PSC and IAC, only a limited number of patient samples were used in this study. However, assuming an AUC of 0.69 for CA19-9 and 0.85 for a useful biomarker panel in the diagnosis of BTC, only 22 patients in each of the malignant/benign groups would be required to detect a 0.16 AUC difference in diagnostic efficacy of the biomarker panel over CA19-9. Furthermore, the success of correctly diagnosing BTC *vs* benign PSC strictures using CA19-9 alone may be as low as 50% at a CA19-9 cutoff of 37 U ml^−1^ (Charatcharoenwitthaya *et al*, 2008). Based on our findings, further validation studies are warranted.

In conclusion, in this preliminary study of circulating biomarkers in patients with benign and malignant biliary strictures, a serum immunodepletion 2D-DIGE/MS strategy was used to identify LRG1 as a novel serum biomarker for detection of BTC. LRG1 had a discriminatory power similar to CA19-9 in differentiating benign from malignant biliary strictures, independent of biliary obstruction and inflammation. LRG1 is overexpressed in CCA cells compared with benign biliary epithelium. Serum IL-6 levels were significantly elevated in BTC compared with benign biliary disease, and a combination of LRG1, CA19-9 and IL-6 strongly discriminates BTC from benign biliary disease. This circulating serum biomarker panel may have important diagnostic implications for BTC, particularly in the context of high-risk groups such as patients with PSC.

## Figures and Tables

**Table 1:** Demographics, clinical and pathological characteristics of BTC patients

Total number of BTC patients	37
Males	23
Females	14
Cholangiocarcinoma	35
Gallbladder carcinoma	2
	
Positive cytology	15
Positive histology	22
Poorly differentiated	11
Moderately differentiated	10
Well differentiated	1
	
Intrahepatic cholangiocarcinoma	2
*Extrahepatic cholangiocarcinoma*	33
Hilar	29
Distal bile duct	4
	
Stage	
T1 or T2	17
T3 or T4	20
	
Patients deceased	32/37 (86%)
Median survival and range (months)	8.4 (1.4–43.9)
Patients alive	5/37 (14%)
Median survival and range (months)	46.1 (36.8–74.1)

Abbreviation: BTC=biliary tract cancer.

**Table 2:** Demographics and biochemical profile of patient cohort used for 2D-DIGE biomarker discovery

	**Clinical group**			
**Variable**	**BTC**	**PSC**	**IAC**	**Healthy**
Number of patients	37	11	7	30
Female : male	11:23	3:8	0:7	12:18
Age (years)	69 (27–93)	48 (22–76)	64 (43–71)	64 (40–79)
Bilirubin (*μ*mol l^−1^)	40 (8–616)	20 (7–457)	12 (5–40)	—
CA19-9 (U ml^−1^)	299 (1–145528)	20 (1–4119)	15 (1–52)	—
CA19-9 >37 U ml^−1^	30/37	4/11	1/4	—
CRP mg l^−1^ (*N*<5)	44.4 (1–171)	9.9 (1–194.2)	8.6 (5–35.7)	—
WCC ( × 10^9^ per l)	8.2 (3.3–14.8)	6.0 (4.3–15.0)^a^	7.6 (2.3–9.8)	—
Neutrophils ( × 10^9^ per l)	5.9 (2.3–12.0)	3.3 (2.8–13.4)^a^	4.1 (0.7–5.4)	—
ALP (U l^−1^)	577 (138–1925)	195 (98–514)	229 (69–642)	—
IgG4 g l^−1^ (*N*<1.3 g l^−1^)	—	—	1.5 (0.49–2.57)	—

Abbreviations: ALP=alkaline phosphatase; BTC=biliary tract cancer; CA19-9=carbohydrate antigen 19-9; CRP=C-reactive protein; IAC=IgG4-associated cholangitis; IgG4=immunoglobulin G4; PSC=primary sclerosing cholangitis; WCC=white cell count; 2D-DIGE=two-dimensional difference gel electrophoresis.

Unless otherwise indicated, values indicate median (range).

a WCC and neutrophil counts were available for only 6 of the 11 PSC patients.

**Table 3:** Identification of putative biomarkers from 2D-DIGE discovery experiment

**Protein name**	**IPI accession no.**	**Mascot score**	**Sequence coverage (%)**	**No. peptides**	**BTC *vs* healthy (average ratio)**	***P*-value**	**BTC *vs* PSC (Av ratio)**	***P*-value**
Apolipoprotein E	IPI00021842	102	22	10	1.76	0.02	2.02	0.01
α-1-anti-chymotrypsin, isoform 1	IPI00847635	2274	52	225	5.07	<0.0001	3.88	<0.0001
α-1-antitrypsin, isoform 1	IPI00553177	665	70	144	8.05	<0.0001	3.42	<0.0001
Haptoglobin	IPI00431645	567	41	69	3.5	<0.0001	5.14	<0.0001
Heat-shock 90 kDa protein 1α isoform 1	IPI00382470	100	11	12	−2.88	<0.0001	−3.07	<0.0001
LRG1	IPI00022417	195	19	21	4.49	<0.0001	3.57	<0.0001
Mannan-binding lectin serine protease 2	IPI00306378	76	45	30	3.01	0.007	2.59	0.0095
Tetranectin/CLEC3B	IPI00009028	107	68	22	−1.97	<0.0001	−1.5	0.0095
Vimentin	IPI00418471	927	54	84	4.29	<0.0001	2.17	0.001

Abbreviations: BTC=biliary tract cancer; LRG1=leucine-rich α-2-glycoprotein; PIP=international protein index; PSC=primary sclerosing cholangitis; 2D-DIGE=two-dimensional difference gel electrophoresis.

Identifications were based on liquid chromatography-tandem mass spectrometry analysis and database searching according to Materials and Methods.

**Table 4:** Demographics and distribution of serum proteins in patient cohort used for serum LRG1 verification

	**Biliary tract cancer**	**Benign biliary disease**	***P-*value**	**Healthy volunteers**	***P-*value**
Males : females	18 : 13	10 : 3	0.32^a^	10 : 5	0.75^a^
Patient age (years)	71 (66–78)	50 (40–58)	< 0.0001	63 (50–74)	0.05
Bilirubin (*μ*mol l^−1^)	40 (24–225)	20 (9–54)	0.04	—	—
ALP (U l^−1^)	626 (244–902)	151 (114–325)	0.0002	—	—
CRP (mg l^−1^)	45.0 (19.8–91.2)	9.6 (1.2–30.3)	0.006	—	—
WCC ( × 10^9^ per l)	8.5 (6.0–11.0)	6.3 (5.1–7.5)	0.09	—	—
Neutrophils ( × 10^9^ per l)	5.9 (4.4–8.8)	3.7 (3.0–4.4)	0.02	—	—
CA19-9 (U ml^−1^)	304 (129–2135)	20 (9–59)	0.0005	—	—
LRG1 (*μ*g ml^−1^)	67.4 (58.6–83.4)	41.8 (32.9–53.4)	0.0001	25.0 (18.7–30.4)	<0.0001

Abbreviations: ALP=alkaline phosphatase; CA19-9=carbohydrate antigen 19-9; CRP=C-reactive protein; LRG1=leucine-rich *α*-2-glycoprotein; WCC=white cell count.

Unless otherwise indicated, values are median (interquartile range) and *P*-values are obtained from Mann–Whitney *U*-test for continuous data. Comparisons were made between distribution of values in the biliary tract cancer group compared with benign biliary disease (primary sclerosing cholangitis (*n*=11)/IgG4-associated cholangitis (*n*=2)) and healthy volunteer groups.

a *P*-values are obtained using Fisher's exact test.
