# Supplementary material for: A combination of serum leucine-rich α-2-glycoprotein 1, CA19-9 and interleukin-6 differentiate biliary tract cancer from benign biliary strictures
Source: Br J Cancer. 2011 Oct 4;105(9):1370–8. doi: 10.1038/bjc.2011.376 (PMC3241550; doi:10.1038/bjc.2011.376)
Supplement: Supplementary Figures [file bjc2011376x1.pdf]

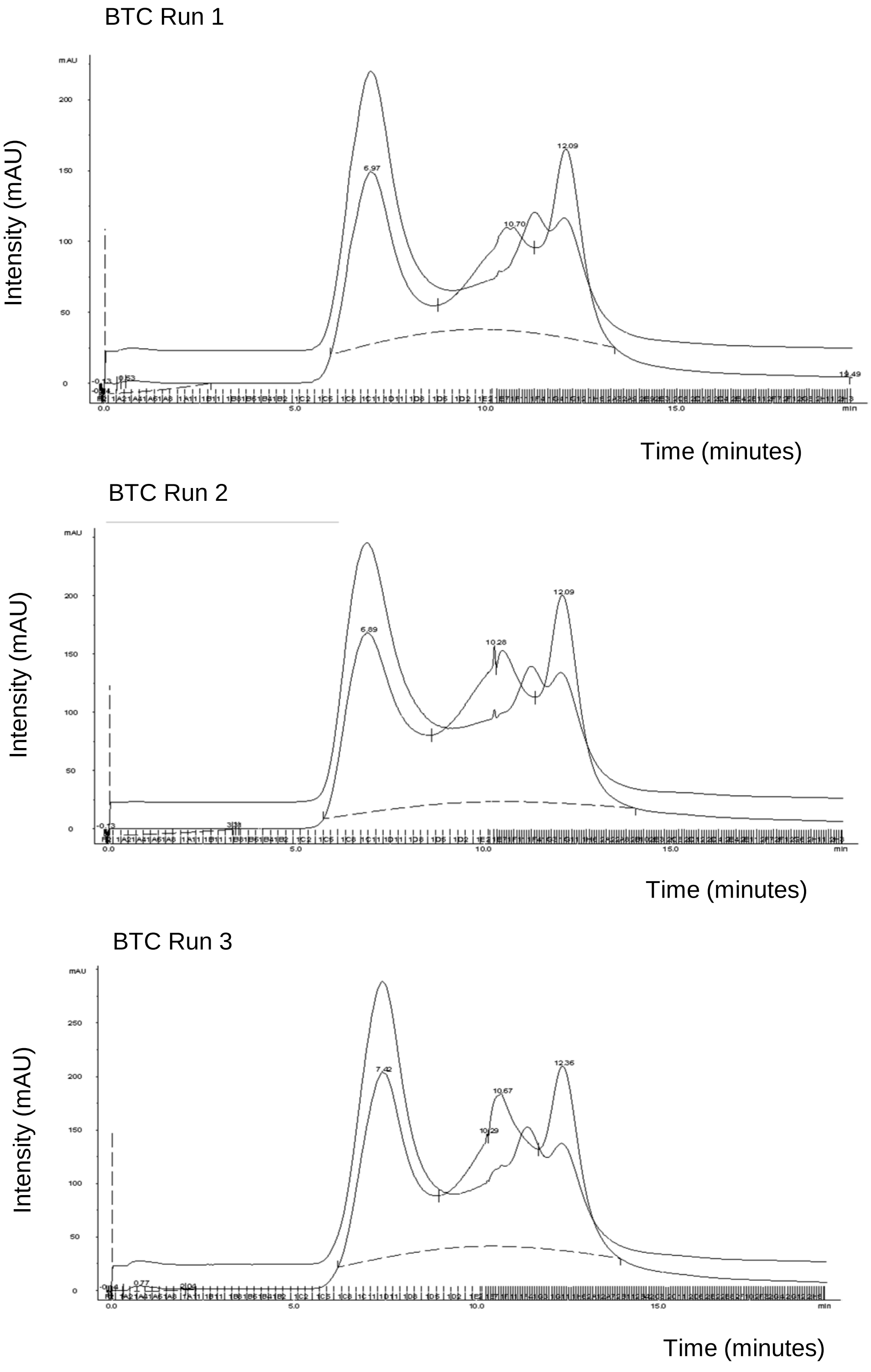

Figure S1. Liquid chromatograms for three run injections of crude diluted pooled BTC serum using immunoaffinity antibody columns coupled to fast protein liquid chromatography (FPLC).

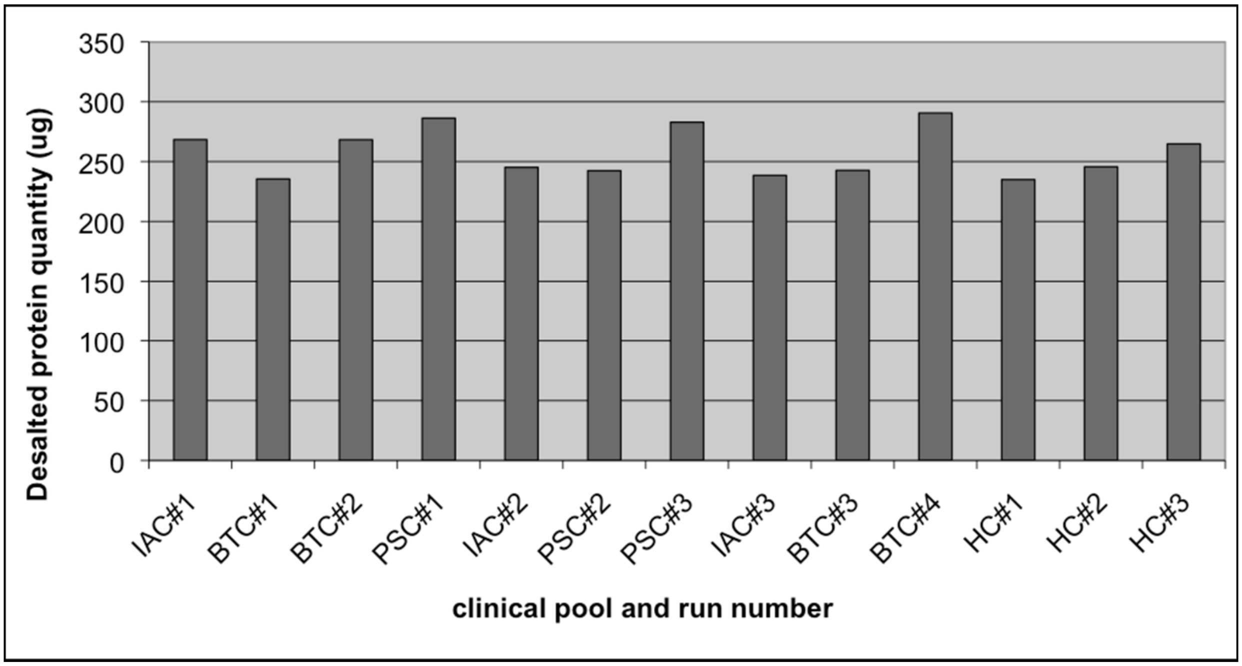

Figure S2. Quantity of desalted and concentrated protein recovered following FPLC immunoaffinity depletion and spin filter centrifugation for each of the clinical group runs.
