# Supplementary material for: A combination of serum leucine-rich α-2-glycoprotein 1, CA19-9 and interleukin-6 differentiate biliary tract cancer from benign biliary strictures
Source: Br J Cancer. 2011 Oct 4;105(9):1370–8. doi: 10.1038/bjc.2011.376 (PMC3241550; doi:10.1038/bjc.2011.376)
Supplement: Supplementary Table S1 [file bjc2011376x2.pdf]

Spot no.	Protein Name	IPI	Mascot score	Sequence coverage (%)	No. peptides	MW pred	pI pred	MW expt	pI expt	BTC vs HC Av. Ratio	t-test	BTC vs PSC Av ratio	t-test	BTC vs IAC Av ratio	t-test	PSC vs HC Av. Ratio	t-test	PSC vs IAC Av. Ratio	t-test
595	Alpha-2-macroglobulin	IPI00478003	176	19	45	164600	6	78353	7.34	-1.94	0.00011	-1.93	7.30E-05	-1.80	6.70E-06	-1.01	0.92	1.07	0.12
595	Gelsolin, isoform 1	IPI00026314	33	8	11	86043	5.9	78353	7.34	-1.94	0.00011	-1.93	7.30E-05	-1.80	6.70E-06	-1.01	0.92	1.07	0.12
597	Alpha-2-macroglobulin	IPI00478003	841	30	71	164600	6	78079	7.51	-2.05	0.0045	-1.95	0.00021	-1.79	3.40E-05	-1.05	0.78	1.09	0.12
597	Complement factor B, highly similar to	IPI00019591	126	9	19	143191	6.82	78079	7.51	-2.05	0.0045	-1.95	0.00021	-1.79	3.40E-05	-1.05	0.78	1.09	0.12
597	Gelsolin, isoform 1	IPI00026314	56	10	10	86043	5.9	78079	7.51	-2.05	0.0045	-1.95	0.00021	-1.79	3.40E-05	-1.05	0.78	1.09	0.12
609	Alpha-2-macroglobulin	IPI00478003	689	17	51	164600	6	77738	5.67	-2.4	0.0014	-1.78	0.0098	-1.80	0.0051	-1.35	0.016	-1.01	0.82
609	Keratin, type II cytoskeletal 2 epidermal	IPI00021304	124	10	6	66111	8.07	77738	5.67	-2.4	0.0014	-1.78	0.0098	-1.80	0.0051	-1.35	0.016	-1.01	0.82
609	Gelsolin, isoform 1	IPI00026314	64	12	16	86043	5.9	77738	5.67	-2.4	0.0014	-1.78	0.0098	-1.80	0.0051	-1.35	0.016	-1.01	0.82
609	Long-chain fatty acid CoA ligase 6, isoform 3	IPI00296333	40	2	5	78704	6.98	77738	5.67	-2.4	0.0014	-1.78	0.0098	-1.80	0.0051	-1.35	0.016	-1.01	0.82
609	SERPIND1; Serpin peptidase inhibitor clade D, member 1	IPI00292950	38	8	5	60368	6.72	77738	5.67	-2.4	0.0014	-1.78	0.0098	-1.80	0.0051	-1.35	0.016	-1.01	0.82
609	Galectin-3-binding protein	IPI00023673	34	4	3	66202	5.13	77738	5.67	-2.4	0.0014	-1.78	0.0098	-1.80	0.0051	-1.35	0.016	-1.01	0.82
637	Gelsolin, isoform 1	IPI00026314	717	20	74	86043	5.9	86000	6.7	-2.28	7.30E-05	-1.88	0.00038	-2.01	8.10E-05	-1.21	0.014	-1.07	0.16
637	Keratin, type I cytoskeletal 10	IPI00009865	173	17	15	59046	5.09	86000	6.7	-2.28	7.30E-05	-1.88	0.00038	-2.01	8.10E-05	-1.21	0.014	-1.07	0.16
637	Complement C2 (Fragment)	IPI00303963	68	7	6	84583	7.23	86000	6.7	-2.28	7.30E-05	-1.88	0.00038	-2.01	8.10E-05	-1.21	0.014	-1.07	0.16
654	Gelsolin, isoform 1	IPI00026314	665	18	62	86043	5.9	76724	4.39	-2.03	0.0019	-1.3	0.079	-1.50	0.012	-1.57	0.0032	-1.15	0.087
654	Keratin, type II cytoskeletal 2 epidermal	IPI00021304	287	17	14	66111	8.07	76724	4.39	-2.03	0.0019	-1.3	0.079	-1.50	0.012	-1.57	0.0032	-1.15	0.087
656	Serotransferrin	IPI00022463	977	30	72	79280	6.81	76926	4.32	-1.61	0.00037	-1.15	0.083	-1.30	0.00027	-1.4	0.0095	-1.13	0.11
656	IgM heavy chain 52 kDa protein	IPI00477090	173	14	13	52449	6.05	76926	4.32	-1.61	0.00037	-1.15	0.083	-1.30	0.00027	-1.4	0.0095	-1.13	0.11
656	Ig mu heavy chain disease protein	IPI00385264	89	13	10	43543	5.13	76926	4.32	-1.61	0.00037	-1.15	0.083	-1.30	0.00027	-1.4	0.0095	-1.13	0.11
676	Keratin, type II cytoskeletal 2 epidermal	IPI00021304	485	33	25	66110	8.07	76926	7.1	-2.88	0.00082	-3.07	0.00095	-1.99	0.0047	1.06	0.38	1.54	0.0033
676	Heat shock 90kDa protein 1, alpha isoform 1	IPI00382470	100	11	12	98670	5.07	74997	7.1	-2.88	0.00082	-3.07	0.00095	-1.99	0.0047	1.06	0.38	1.54	0.0033
752	Serotransferrin	IPI00022463	867	33	68	79280	6.81	72417	9.55	-2.4	0.0013	-2.52	0.0015	-1.58	0.013	1.05	0.45	1.6	0.0009
754	Serotransferrin	IPI00022463	739	31	64	79280	6.81	72038	8.09	-2.48	0.00031	-3.94	0.00026	-2.11	0.0005	1.59	0.0076	1.87	0.0021
754	IgM heavy chain 52 kDa protein	IPI00477090	407	22	27	52449	6.05	72417	8.09	-2.48	0.00031	-3.94	0.00026	-2.11	0.0005	1.59	0.0076	1.87	0.0021
754	Ig mu heavy chain disease protein	IPI00385264	116	30	21	43543	5.13	72417	8.09	-2.48	0.00031	-3.94	0.00026	-2.11	0.0005	1.59	0.0076	1.87	0.0021
754	Lumican	IPI00020986	46	9	3	38747	6.16	72417	8.09	-2.48	0.00031	-3.94	0.00026	-2.11	0.0005	1.59	0.0076	1.87	0.0021
755	Serotransferrin	IPI00022463	1348	34	81	79280	6.81	72290	7.89	-3.34	0.0014	-6.07	0.00031	-2.85	0.0023	1.82	0.00014	2.13	1.60E-05
755	IgM heavy chain 52 kDa protein	IPI00477090	364	25	27	52449	6.05	72290	7.89	-3.34	0.0014	-6.07	0.00031	-2.85	0.0023	1.82	0.00014	2.13	1.60E-05
793	Serotransferrin	IPI00022463	1198	68	178	79280	6.81	79000	7.8	-2.77	0.00031	-2.72	0.011	-2.18	0.0013	-1.02	0.79	1.25	0.42
804	SERPIND1, Serpin peptidase inhibitor clade D, member 1	IPI00292950	335	36	47	60368	6.72	69681	6.46	-1.75	0.00014	-2.76	6.80E-06	-2.17	6.30E-06	1.58	0.00084	1.27	0.0043
804	Complement C2 (Fragment)	IPI00303963	130	26	36	84583	7.23	69681	6.46	-1.75	0.00014	-2.76	6.80E-06	-2.17	6.30E-06	1.58	0.00084	1.27	0.0043
804	C4b-binding protein alpha chain	IPI00021727	38	12	10	69042	7.15	69681	6.46	-1.75	0.00014	-2.76	6.80E-06	-2.17	6.30E-06	1.58	0.00084	1.27	0.0043
872	SERPINA3; highly similar to	IPI00550991	2275	49	225	50737	5.42	66639	3.6	5.07	0.00055	3.88	0.00043	2.13	0.0039	1.31	0.061	-1.82	3.60E-05
872	SERPINA3; Alpha-1-antichymotrypsin, isoform 1	IPI00847635	2274	52	225	47792	5.33	66639	3.6	5.07	0.00055	3.88	0.00043	2.13	0.0039	1.31	0.061	-1.82	3.60E-05
872	Cell cycle checkpoint protein RAD1, isoform 1	IPI00003647	36	2	36	32434	4.73	66639	3.6	5.07	0.00055	3.88	0.00043	2.13	0.0039	1.31	0.061	-1.82	3.60E-05
920	SERPINA3; Alpha-1-antichymotrypsin, isoform 1	IPI00847635	2287	59	304	50737	5.42	64628	3.71	2.52	0.017	2.25	0.0077	1.43	0.087	1.12	0.5	-1.57	0.0021
920	Mannan-binding lectin serine protease 1, isoform 1	IPI00299307	139	21	20	80849	5.29	64628	3.71	2.52	0.017	2.25	0.0077	1.43	0.087	1.12	0.5	-1.57	0.0021
920	LCAT Phosphatidylcholine-sterol acyltransferase	IPI00022331	55	15	6	49888	5.71	64628	3.71	2.52	0.017	2.25	0.0077	1.43	0.087	1.12	0.5	-1.57	0.0021
920	Lumican	IPI00020986	48	5	4	38747	6.16	64628	3.71	2.52	0.017	2.25	0.0077	1.43	0.087	1.12	0.5	-1.57	0.0021
920	PLXNA1 Conserved hypothetical protein	IPI00749037	37	9	2	10788	9.3	64628	3.71	2.52	0.017	2.25	0.0077	1.43	0.087	1.12	0.5	-1.57	0.0021
921	SERPINA3; Alpha-1-antichymotrypsin, isoform 1	IPI00847635	1678	62	254	47792	5.33	64346	3.75	2.56	0.00075	1.9	0.00045	1.39	0.0047	1.35	0.049	-1.37	0.01
921	Alpha-2-macroglobulin	IPI00478003	131	3	5	164600	6	64346	3.75	2.56	0.00075	1.9	0.00045	1.39	0.0047	1.35	0.049	-1.37	0.01
921	Mannan-binding lectin serine protease 1, isoform 1	IPI00299307	78	19	13	80849	5.29	64346	3.75	2.56	0.00075	1.9	0.00045	1.39	0.0047	1.35	0.049	-1.37	0.01
921	Lumican	IPI00020986	57	28	10	38747	6.16	64346	3.75	2.56	0.00075	1.9	0.00045	1.39	0.0047	1.35	0.049	-1.37	0.01
921	Vitronectin	IPI00298971	52	3	5	55069	5.55	64346	3.75	2.56	0.00075	1.9	0.00045	1.39	0.0047	1.35	0.049	-1.37	0.01
921	SERPINA6; Corticosteroid-binding globulin	IPI00027482	33	8	4	45283	5.64	64346	3.75	2.56	0.00075	1.9	0.00045	1.39	0.0047	1.35	0.049	-1.37	0.01
1031	SERPINA1; Alpha-1-antitrypsin, isoform 1	IPI00553177	665	70	144	46878	5.37	55000	3.9	8.05	3.00E-05	3.42	8.60E-06	6.02	1.40E-05	2.35	0.00076	1.76	0.00075
1031	Keratin, type I cytoskeletal 10	IPI00009865	242	18	19	59046	5.09	55000	3.9	8.05	3.00E-05	3.42	8.60E-06	6.02	1.40E-05	2.35	0.00076	1.76	0.00075
1031	Keratin, type I cytoskeletal 9	IPI00019359	194	22	24	62255	5.14	55000	3.9	8.05	3.00E-05	3.42	8.60E-06	6.02	1.40E-05	2.35	0.00076	1.76	0.00075
1031	SERPINA7; Thyroxine-binding globulin	IPI00292946	87	16	7	46637	5.87	55000	3.9	8.05	3.00E-05	3.42	8.60E-06	6.02	1.40E-05	2.35	0.00076	1.76	0.00075
1031	RPP30 Ribonuclease P	IPI00478229	62	2	2	38591	9.78	55000	3.9	8.05	3.00E-05	3.42	8.60E-06	6.02	1.40E-05	2.35	0.00076	1.76	0.00075
1031	SERPINA6; Corticosteroid-binding globulin	IPI00027482	53	5	6	45283	5.64	55000	3.9	8.05	3.00E-05	3.42	8.60E-06	6.02	1.40E-05	2.35	0.00076	1.76	0.00075
1035	Vimentin	IPI00418471	927	54	84	53676	5.06	58747	4.22	4.29	5.90E-05	2.17	0.0012	2.24	0.00054	1.98	0.00045	1.03	0.67
1035	SERPINA1; Alpha-1-antitrypsin, isoform 1	IPI00553177	618	69	112	46878	5.37	58747	4.22	4.29	5.90E-05	2.17	0.0012	2.24	0.00054	1.98	0.00045	1.03	0.67
1035	Monocyte differentiation antigen CD14	IPI00029260	69	10	5	40678	5.84	58747	4.22	4.29	5.90E-05	2.17	0.0012	2.24	0.00054	1.98	0.00045	1.03	0.67
1035	Tubulin alpha-4A chain	IPI00007750	56	7	9	50634	4.95	58747	4.22	4.29	5.90E-05	2.17	0.0012	2.24	0.00054	1.98	0.00045	1.03	0.67
1052	SERPINA1; Alpha-1-antitrypsin, isoform 1	IPI00553177	1234	71	209	46878	5.37	57576	4.06	3.01	0.0008	1.6	0.047	1.62	0.08	1.88	0.014	1.01	0.92
1052	HAUS augmin-like complex subunit 3	IPI00029372	37	3	7	70006	5.4	57576	4.06	3.01	0.0008	1.6	0.047	1.62	0.08	1.88	0.014	1.01	0.92
1071	SERPINA1; Alpha-1-antitrypsin, isoform 1	IPI00553177	672	52	86	46878	5.37	56726	4.3	2.16	0.005	1.21	0.39	1.32	0.12	1.79	0.037	1.09	0.75
1072	SERPINA1; Alpha-1-antitrypsin, isoform 1	IPI00553177	558	43	70	46878	5.37	56527	4.07	4.77	4.50E-05	3.15	3.40E-05	4.03	8.40E-05	1.52	0.0034	1.28	0.026
1076	SERPINA1; Alpha-1-antitrypsin, isoform 1	IPI00553177	589	80	34	46878	5.37	56875	4.41	2.57	0.00018	-1.05	0.6	1.20	0.041	2.7	0.00034	1.26	0.046
1076	Apolipoprotein A-IV	IPI00304273	123	31	16	45371	5.28	56875	4.41	2.57	0.00018	-1.05	0.6	1.20	0.041	2.7	0.00034	1.26	0.046
1076	SERPINF1; Pigment epithelium-derived factor	IPI00006114	68	7	5	46484	5.97	56875	4.41	2.57	0.00018	-1.05	0.6	1.20	0.041	2.7	0.00034	1.26	0.046
1131	IgG heavy chain	IPI00930124	441	19	43	52829	7.51	54918	8.13	1.44	0.0028	-1.67	0.00016	-2.25	9.50E-06	2.4	8.40E-05	-1.35	0.00032
1131	IgG4 chain C region	IPI00829814	228	21	35	35940	7.18	54918	8.13	1.44	0.0028	-1.67	0.00016	-2.25	9.50E-06	2.4	8.40E-05	-1.35	0.00032
1132	IgG heavy chain	IPI00930124	243	16	31	52829	7.51	52000	8.1	1.51	0.0056	-1.71	9.30E-06	-2.41	3.00E-07	2.58	0.00026	-1.41	8.90E-05
1132	IgG4 chain C region	IPI00829814	124	11	32	35940	7.18	52000	8.1	1.51	0.0056	-1.71	9.30E-06	-2.41	3.00E-07	2.58	0.00026	-1.41	8.90E-05
1132	Glutamate dehydrogenase 1, mitochondrial	IPI00016801	113	12	15	61701	7.66	52000	8.1	1.44	0.0028	-1.67	0.00016	-2.25	3.00E-07	2.4	8.40E-05	-1.35	0.00032
1144	Leucine-rich alpha-2-glycoprotein	IPI00022417	395	24	38	38382	6.45	53965	3.55	4.49	4.90E-05	3.57	6.00E-05	2.95	2.40E-05	1.26	0.061	-1.21	0.038
1168	Leucine-rich alpha-2-glycoprotein	IPI00022417	195	19	21	38382	6.45	53075	3.6	3.4	9.30E-06	2.88	6.40E-05	2.36	3.00E-05	1.18	0.037	-1.22	0.018
1168	Keratin, type I cytoskeletal 9	IPI00019359	138	8	5	62255	5.14	53075	3.6	3.4	9.30E-06	2.88	6.40E-05	2.36	3.00E-05	1.18	0.037	-1.22	0.018

1168	Apolipoprotein A-IV	IPI00304273	71	22	12	45371	5.28	53075	3.6	3.4	9.30E-06	2.88	6.40E-05	2.36	3.00E-05	1.18	0.037	-1.22	0.018
1174	Leucine-rich alpha-2-glycoprotein	IPI00022417	145	18	18	38382	6.45	52337	3.77	4.67	3.10E-05	3.5	0.0011	2.06	0.00026	1.33	0.15	-1.7	0.019
1174	HP protein; haptoglobin	IPI00431645	94	19	19	31647	8.48	52337	3.77	4.67	3.10E-05	3.5	0.0011	2.06	0.00026	1.33	0.15	-1.7	0.019
1178	Leucine-rich alpha-2-glycoprotein	IPI00022417	168	14	18	38382	6.45	50000	3.5	3.06	8.40E-05	2.52	0.00033	2.14	0.00019	1.22	0.059	-1.18	0.055
1183	HP protein; haptoglobin	IPI00431645	458	49	68	31647	8.48	51159	3.87	7.4	2.00E-05	5.81	0.0003	2.00	0.00049	1.27	0.2	-2.91	0.0014
1183	Leucine-rich alpha-2-glycoprotein	IPI00022417	165	46	24	38382	6.45	51159	3.87	7.4	2.00E-05	5.81	0.0003	2.00	0.00049	1.27	0.2	-2.91	0.0014
1183	POTE Ankyrin domain family member J	IPI00738655	41	3	4	118740	5.66	51159	3.87	7.4	2.00E-05	5.81	0.0003	2.00	0.00049	1.27	0.2	-2.91	0.0014
1185	Leucine-rich alpha-2-glycoprotein	IPI00022417	168	14	18	38382	6.45	51293	3.7	2.32	8.10E-05	2.39	0.0005	1.94	0.00023	-1.03	0.66	-1.23	0.038
1197	Leucine-rich alpha-2-glycoprotein	IPI00022417	450	28	36	38382	6.45	50580	3.76	1.69	0.00034	1.54	0.0038	1.44	0.0022	1.09	0.23	-1.07	0.36
1197	Alpha-2-glycoprotein 1, zinc	IPI00940069	143	23	11	34465	5.71	50580	3.76	1.69	0.00034	1.54	0.0038	1.44	0.0022	1.09	0.23	-1.07	0.36
1221	Keratin, type II cytoskeletal 1	IPI00220327	276	24	21	66170	8.15	48287	4.1	4.59	0.00076	8.17	0.00029	1.47	0.015	-1.78	0.063	-5.56	0.00065
1226	HP protein; haptoglobin	IPI00431645	567	41	69	31647	8.48	48626	4.43	3.5	0.00093	5.14	2.90E-05	1.40	0.022	-1.47	0.059	-3.67	0.00011
1230	HP protein; haptoglobin	IPI00431645	183	22	31	31647	8.48	48372	3.75	2.81	1.60E-05	2.03	0.00041	2.19	0.0005	1.38	0.0045	1.08	0.43
1248	HP protein; haptoglobin	IPI00431645	995	54	190	31647	8.48	45000	4.1	3	0.0003	4.83	4.00E-05	1.33	0.019	-1.61	0.0053	-3.63	3.60E-05
1248	Haptoglobin-related protein, isoform 1	IPI00477597	559	26	81	39496	6.42	45000	4.1	3	0.0003	4.83	4.00E-05	1.33	0.019	-1.61	0.0053	-3.63	3.60E-05
1258	Haptoglobin-related protein 47 kDa protein	IPI00641737	593	29	68	47378	6.28	46463	4.69	2.42	0.00048	4.12	8.50E-05	1.17	0.13	-1.7	0.0041	-3.51	0.00015
1258	HP protein; haptoglobin	IPI00431645	593	39	67	31647	8.48	46463	4.69	2.42	0.00048	4.12	8.50E-05	1.17	0.13	-1.7	0.0041	-3.51	0.00015
1261	HP protein; haptoglobin	IPI00431645	134	16	8	31647	8.48	46585	4.97	1.68	0.0014	2.38	7.80E-05	-1.16	0.011	-1.42	0.0086	-2.77	2.40E-05
1262	HP protein; haptoglobin	IPI00431645	401	49	54	31647	8.48	46219	5.38	1.25	0.03	1.25	0.013	-1.83	3.10E-06	1.01	0.93	-2.29	0.00011
1262	Sex hormone-binding globulin, isoform 1	IPI00023019	91	9	5	43980	6.22	46219	5.38	1.25	0.03	1.25	0.013	-1.83	3.10E-06	1.01	0.93	-2.29	0.00011
1262	Mitochondrial tumor suppressor 1, isoform 3	IPI00102820	33	4	2	50798	8.52	46219	5.38	1.25	0.03	1.25	0.013	-1.83	3.10E-06	1.01	0.93	-2.29	0.00011
1264	Haptoglobin, isoform 2 preproprotein	IPI00478493	415	39	65	38941	6.13	45736	5.85	-1.13	0.48	-1.5	0.039	-3.32	0.0019	1.33	0.13	-2.21	0.0008
1278	HP protein; haptoglobin	IPI00431645	560	35	83	31647	8.48	45021	6.33	-1.05	0.69	-1.44	0.11	-3.61	0.0017	1.36	0.03	-2.51	0.00025
1289	HP protein; haptoglobin	IPI00431645	182	48	32	31647	8.48	44201	4.68	3.45	0.00079	3.88	0.00016	1.28	0.046	-1.13	0.42	-3.04	3.90E-05
1289	Keratin, type II cytoskeletal 1	IPI00220327	71	12	9	66170	8.15	44201	4.68	3.45	0.00079	3.88	0.00016	1.28	0.046	-1.13	0.42	-3.04	3.90E-05
1291	Keratin, type II cytoskeletal 2 epidermal	IPI00021304	240	32	31	66111	8.07	44124	6.82	-1.01	0.81	-1.56	0.073	-3.95	0.0018	1.55	0.0017	-2.53	8.20E-05
1291	HP protein; haptoglobin	IPI00431645	186	50	42	31647	8.48	44124	6.82	-1.01	0.81	-1.56	0.073	-3.95	0.0018	1.55	0.0017	-2.53	8.20E-05
1300	HP protein; haptoglobin	IPI00431645	320	58	50	31647	8.48	43207	4.99	2.7	0.0029	3.94	0.00072	1.37	0.059	-1.46	0.052	-2.87	0.00043
1309	Haptoglobin, isoform 2 preproprotein	IPI00478493	230	49	45	38941	6.13	42718	5.4	2.3	0.003	2.73	0.0022	1.10	0.4	-1.19	0.29	-2.48	0.0013
1322	Apolipoprotein E	IPI00021842	447	71	66	36246	5.65	40996	4.64	2.36	0.00027	1.14	0.13	1.60	0.0035	2.07	0.00062	1.4	0.013
1322	Clusterin, isoform 1	IPI00291262	61	12	9	53031	5.89	40996	4.64	2.36	0.00027	1.14	0.13	1.60	0.0035	2.07	0.00062	1.4	0.013
1322	Arfaptin-1, isoform B	IPI00021258	44	5	3	41770	6.24	40996	4.64	2.36	0.00027	1.14	0.13	1.60	0.0035	2.07	0.00062	1.4	0.013
1329	Apolipoprotein E	IPI00021842	322	74	55	36246	5.65	40249	4.83	2.62	0.00043	1.32	0.058	1.91	0.0024	1.98	0.00029	1.45	0.0046
1329	Clusterin, isoform 1	IPI00291262	47	7	3	53031	5.89	40249	4.83	2.62	0.00043	1.32	0.058	1.91	0.0024	1.98	0.00029	1.45	0.0046
1338	Apolipoprotein E	IPI00021842	341	73	51	36246	5.65	39001	4.84	1.97	0.0044	-1.06	0.5	3.05	0.00011	2.09	0.0026	3.23	4.70E-05
1338	Twinfilin-2	IPI00550917	39	5	4	39751	6.37	39001	4.84	1.97	0.0044	-1.06	0.5	3.05	0.00011	2.09	0.0026	3.23	4.70E-05
1353	Apolipoprotein E	IPI00021842	102	22	10	36246	5.65	37200	7.68	1.76	0.023	2.02	0.012	2.50	0.0048	-1.15	0.088	1.24	0.051
1353	Ficolin-3; isoform 1	IPI00293925	59	6	4	33395	6.2	37200	7.68	1.76	0.023	2.02	0.012	2.50	0.0048	-1.15	0.088	1.24	0.051
1353	Complement component 2	IPI00515098	49	16	4	13497	9.46	37200	7.68	1.76	0.023	2.02	0.012	2.50	0.0048	-1.15	0.088	1.24	0.051
1391	Carbonic anhydrase 1	IPI00215983	483	57	51	28909	6.59	31363	8.13	-1.1	0.31	-1.3	0.046	1.55	0.0047	1.18	0.11	2.02	0.00065
1405	Apolipoprotein A-I	IPI00021841	142	65	48	30759	5.56	30363	4.43	-1.79	0.0068	1.38	0.054	-1.39	0.039	-2.48	3.10E-05	-1.92	5.80E-05
1405	Ig lambda chain	IPI00827875	60	20	7	25038	7.55	30363	4.43	-1.79	0.0068	1.38	0.054	-1.39	0.039	-2.48	3.10E-05	-1.92	5.80E-05
1405	C-reactive protein, isoform	IPI00022389	50	20	9	25194	5.45	30363	4.43	-1.79	0.0068	1.38	0.054	-1.39	0.039	-2.48	3.10E-05	-1.92	5.80E-05
1416	Apolipoprotein A-I	IPI00021841	482	82	116	30759	5.56	27000	4.7	-2.29	0.0017	-1.59	0.023	-2.39	0.0011	-1.44	0.02	-1.5	0.01
1416	IgG light chain	IPI00154742	117	29	9	25119	5.93	27000	4.7	-2.29	0.0017	-1.59	0.023	-2.39	0.0011	-1.44	0.02	-1.5	0.01
1443	25 kDa protein	IPI00940069	266	47	51	25721	6.3	28509	9.39	1.54	0.00031	-1.29	0.0067	-1.19	0.012	1.98	7.20E-05	1.08	0.18
1443	Ig kappa chain	IPI00784865	254	47	47	26042	5.94	28509	9.39	1.54	0.00031	-1.29	0.0067	-1.19	0.012	1.98	7.20E-05	1.08	0.18
1443	Putative uncharacterized protein	IPI00550731	215	49	47	26503	8.24	28509	9.39	1.54	0.00031	-1.29	0.0067	-1.19	0.012	1.98	7.20E-05	1.08	0.18
1469	Tetranectin/CLEC3B, isoform	IPI00792115	107	68	22	18125	4.96	21848	5.23	-1.97	0.00034	-1.48	0.0026	-1.40	0.0059	-1.33	0.00015	1.06	0.12
1469	Tetranectin/CLEC3B	IPI00009028	91	56	23	22951	5.52	21848	5.23	-1.97	0.00034	-1.48	0.0026	-1.40	0.0059	-1.33	0.00015	1.06	0.12
1471	Haptoglobin	IPI00641737	130	15	24	47378	6.28	21563	4.85	3.01	0.0069	2.59	0.0095	2.40	0.0023	1.16	0.12	-1.08	0.11
1471	Mannan-binding lectin serine protease 2, isoform 2	IPI00306378	76	45	30	21129	5.61	21563	4.85	3.01	0.0069	2.59	0.0095	2.40	0.0023	1.16	0.12	-1.08	0.11
1471	Tetranectin/CLEC3B, isoform	IPI00792115	63	48	14	18125	4.96	21563	4.85	3.01	0.0069	2.59	0.0095	2.40	0.0023	1.16	0.12	-1.08	0.11
1476	HP protein; haptoglobin	IPI00431645	262	23	42	31647	8.48	30000	5	4.09	3.30E-06	4.6	4.10E-05	1.94	0.0003	-1.12	0.15	-2.37	0.00041
1476	Mannan-binding lectin serine protease 2, isoform 1	IPI00294713	53	8	5	77224	5.47	30000	5	4.09	3.30E-06	4.6	4.10E-05	1.94	0.0003	-1.12	0.15	-2.37	0.00041
1476	Mannan-binding lectin serine protease 2, isoform 2	IPI00306378	45	33	6	21129	5.61	30000	5	4.09	3.30E-06	4.6	4.10E-05	1.94	0.0003	-1.12	0.15	-2.37	0.00041
1480	Haptoglobin	IPI00641737	374	24	70	47378	6.28	18000	6	2.22	0.00034	2.83	0.00014	1.19	0.07	-1.27	0.0044	-2.38	2.40E-05

Table S1 Differentially expressed proteins identified from 2D-DIGE analysis of depleted BTC, PSC, IAC and HC serum samples. Master spot number, protein name, IPI accession number, Mascot protein score, % sequence coverage, number of identified peptides, predicted pI and MW, gel-based MW and pI, and average ratio for each comparison (from triplicates) with associated t-test *P* value are given for each identification. Proteins were identified by LC-MS/MS using the search parameters and results filters stated in Materials and Methods. indicate spots containing two identifications. Orange shading indicates up-regulated proteins and blue shading indicates down-regulated proteins (>2-fold) for each comparison. Green shading separates spot numbers for clarity. Yellow shading indicates isoforms of leucine-rich alpha-2-glycoprotein (LRG1).
