# Supplementary material for: A combination of serum leucine-rich α-2-glycoprotein 1, CA19-9 and interleukin-6 differentiate biliary tract cancer from benign biliary strictures
Source: Br J Cancer. 2011 Oct 4;105(9):1370–8. doi: 10.1038/bjc.2011.376 (PMC3241550; doi:10.1038/bjc.2011.376)
Supplement: Supplementary Information [file bjc2011376x3.pdf]

Supplementary Information - Methods

Patient population and clinical samples

All patients in the BTC group had either cytological confirmation of BTC (15/37) or histology consistent with malignancy (22/37); 11/22 (50%) with positive histology had poorly differentiated adenocarcinoma, 10/22 (45%) had moderately differentiated adenocarcinoma and one patient (5%) had a well-differentiated cancer. Of the BTC patients, 35/37 (95%) had CCA and 2/37 (5%) had gallbladder cancer; 33/35 and 2/35 were extrahepatic and intrahepatic, respectively. 29/33 (88%) of the patients with extrahepatic CCA had lesions located in the liver hilum, whilst the remaining 12% had distal bile duct tumours. Cancers were staged using the TNM staging system (Sobin & Fleming, 1997). T1/2 disease without evidence of nodal or metastatic spread was classified as “early” disease. 17/37 (46%) of patients had early stage disease, whilst 20/37 (54%) had locally advanced or metastatic disease and were classified as “advanced” disease. Median survival in the 33/37 (89%) patients who died of BTC was 8.4 months (range 1.4 to 43.9 months) from time of presentation, whilst 5/37 (14%) patients remained alive at a median of 46 months (range 36 to 74 months). Three of the five patients had cytological or histological confirmation of BTC, while the other two had evidence of disease progression.

FPLC immunoaffinity depletion using tandem IgY14-Supermix system

The columns were primed with filtered and degassed Buffer A (10 mM Tris-HCL, 150 mM NaCl). 110 µL of pooled crude serum was diluted 1 in 5 with Buffer A and filtered through a 0.45 µm spin filter at 10,000g for 60 sec before manual injection. Buffer A was set at a flow rate of 0.5 mL/min for 15 min, then 1.0 mL/min for 10 min to obtain an immuno-depleted flow-through fraction from both columns. The IgY14 and SuperMix columns were regenerated for re-use by separately stripping with 100 mM glycine pH 2.5 at 1.0 mL/min for 7 min, neutralizing with 100 mM Tris-HCL pH 8.0 at 1.0 mL/min for 5 min and then re-equilibrating with Buffer A at 1.0 mL/min for 5 min. Three runs from each pool were performed to obtain sufficient protein for downstream

application. Blank runs with Buffer A were performed between each sample group to clean the columns of residual protein.

Two-dimensional difference gel electrophoresis (2D-DIGE)

Labelling reactions were quenched with a 20-fold molar excess of free L-lysine to dye and left on ice for 10 min. Samples labelled with Cy3 and Cy5 were mixed appropriately and the same amount of Cy2-labelled pool was added to each mixture. Samples were reduced by adding dithiothreitol to 65 mM final concentration. Ampholine/Pharmalyte carriers (1:1 mix, pH 3-10), were added to a final concentration of 2% and bromophenol blue was added. The final volume was adjusted to 450 µL with 2D lysis buffer plus dithiothreitol. For isoelectric focusing, 24 cm, non-linear pH 3-10 IPG strips (GE Healthcare) were rehydrated with labelled samples overnight in the dark at RT and then focussing carried out on a Multiphor II apparatus (GE Healthcare) for a total of 80 kVh at 18℃. Strips were then equilibra ted in 6 M urea, 30 % (v/v) glycerol, 50 mM Tris-HCL pH 6.8 and 2% (w/v) SDS in two steps for 15 min each with gentle rocking, firstly with 65 mM dithiothreitol to reduce disulphide bonds, and secondly with 240 mM iodoacetamide for alkylation. IPG strips were rinsed with electrophoresis buffer (Severn Biotech), transferred onto 1.5 mm 12% SDS-PAGE bonded gels cast between 24 cm low-fluorescence glass plates and overlaid with 0.5% (w/v) low-melting point agarose in electrophoresis buffer with bromophenol blue. Gels were run in an Ettan 12 apparatus (GE Healthcare) at 2 W per gel at 14℃ until the dye front had run off. All steps were carried out in a dedicated clean room. Gel images were obtained by scanning gels between plates on a Typhoon™ 9400 multi-wavelength fluorescence scanner using ImageQuant software (both from GE Healthcare) and image analysis performed using DeCyder™ software V5.0 (GE Healthcare) according to the manufacturer's guidelines. Standardised spot volumes (against Cy2) were calculated and averaged across replicate samples. A Student T-test was performed and spots displaying a ≥1.5 average-fold change in abundance between clinical conditions with *P* values <0.05 were selected for picking. Bonded 2D gels were post-stained with SyproRuby. Post-stained images were imported into DeCyder and matched to the processed Cy-Dye images. Using reference markers, a pick list of coordinates for

protein features of interest was created for automated spot picking on an Ettan automated spot picker (GE Healthcare).

Protein identification by LC-MS/MS

Reversed phase chromatographic separation of peptides was carried out on a 75 μm i.d. x 150 mm C18 PepMap nano LC column of 3 μm bead size and 100 Å pore size (LC Packings) with a linear gradient of 5-50% solvent B (100% acetonitrile + 0.1% formic acid). The mass spectrometer was operated in the data-dependent and positive ion modes. Survey full scan MS spectra (400-2000 m/z) were acquired in the Orbitrap with a resolution of 60,000 at m/z 400 and FT target value of 1 x 10^6 ions. The 6 most abundant ions were selected for CID fragmentation and detected in the ion trap with dynamic exclusion set at 60 sec. For accurate mass measurement, the lock mass option was enabled using the polydimethylcyclosiloxane ion (m/z 455.120025) as an internal calibrant. For searching, MS tolerance was set to 20 ppm and the MS/MS tolerance to 0.8 Da. One missed cleavage was allowed, carbamidomethylation of cysteine was set as a fixed modification and methionine oxidation and deamidated N-term Q were set as variable modifications. Positive protein identifications were accepted when there were at least two unique peptides with ion scores greater than the Mascot homology threshold score at P=0.05.

LRG1 and IL-6 ELISA

Recombinant human LRG1 in the kit was used to construct a standard curve ranging from 0-100 ng/mL. Following trial dilutions in assay buffer, an optimum serum dilution of 1:1000 was used for the final assay which was carried out according to the manufacturer's protocol. The sandwich ELISA was carried out according to the manufacturer's protocol using recombinant human IL-6 in the kit to construct a standard curve ranging from 0-400 pg/mL. A serum dilution of 1:5 was used for PSC/IAC samples and 1:10 for BTC samples. Healthy control samples were not diluted.

LRG1 immunohistochemistry

Immunohistochemistry for LRG was performed on formalin fixed paraffin embedded tissue sectioned at 4µm onto positively charged slides (Superfrost plus, Menzel-Glaser, Germany) using a commercially available affinity purified rabbit polyclonal antibody at a dilution of 1 in 1000 (Cat 13224-1-AP, ProteinTech Chicago, IL, USA). Slides were processed with an automated staining system - the Vision Biosystems BondmaX autostainer (Vision Biosystems, Mount Waverley, Victoria, Australia) used according to manufacturer's protocol and with the manufacturer's retrieval solutions. Heat induced epitope retrieval was performed for 30 minutes in the manufacturer's alkaline retrieval solution ER2 (VBS part no: AR9640). A biotin free detection system was employed (VBS part no: DS 9713) One normal liver biopsy, one gallbladder showing mild chronic cholecystitis, two liver biopsies showing PSC and primary biliary cirrhosis (PBC), and two cholangiocarcinomas were used for the analysis.

Reference

Sobin LH, Fleming ID (1997) TNM Classification of Malignant Tumors, fifth edition (1997). Union Internationale Contre le Cancer and the American Joint Committee on Cancer. *Cancer* **80:** 1803-4
